# Supplementary figures and images for: Microbial Shifts Following Five Years of Cover Cropping and Tillage Practices in Fertile Agroecosystems
Source: Microorganisms. 2020 Nov 11;8(11):1773. doi: 10.3390/microorganisms8111773 (PMC7696634; doi:10.3390/microorganisms8111773)

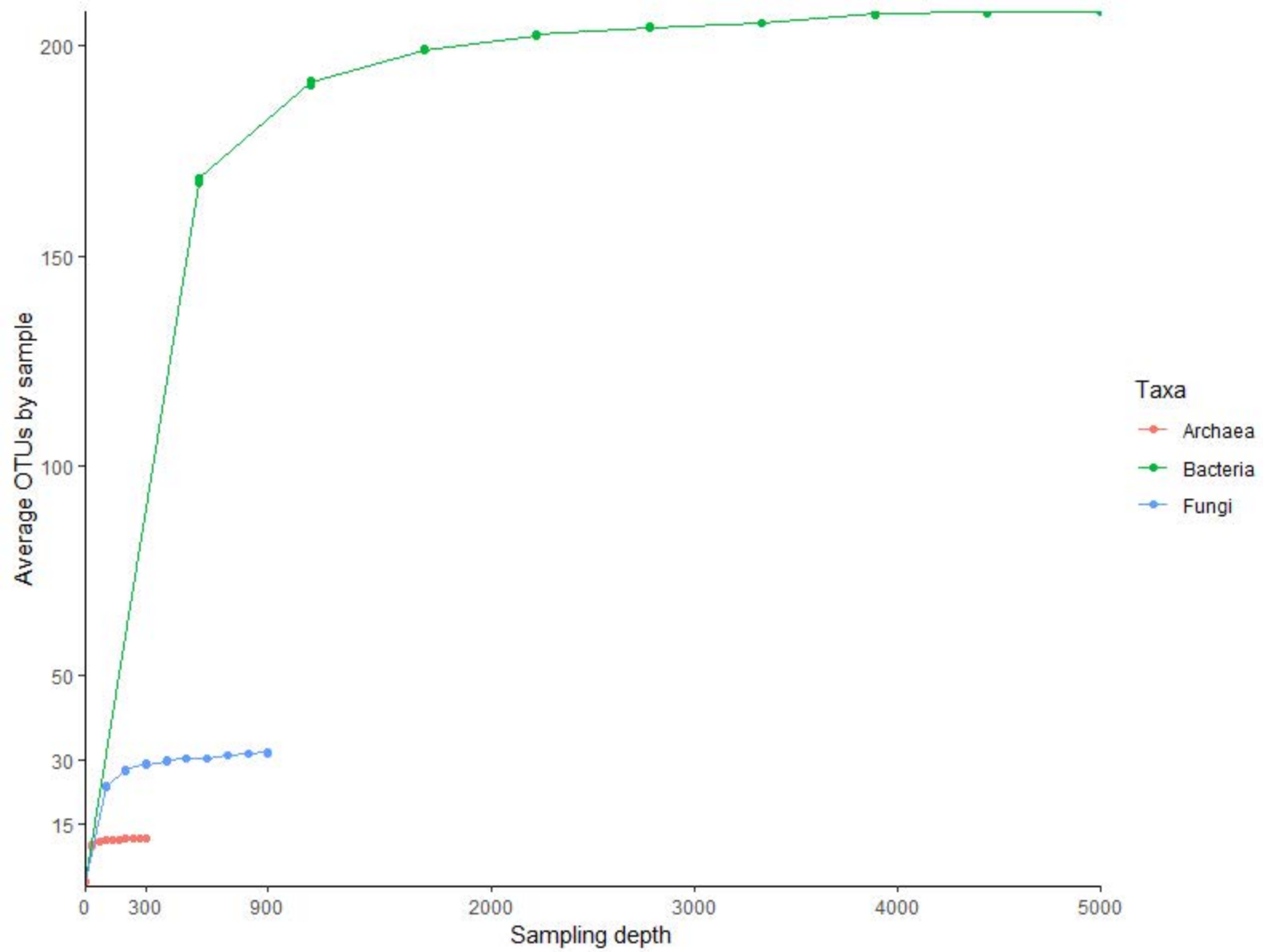

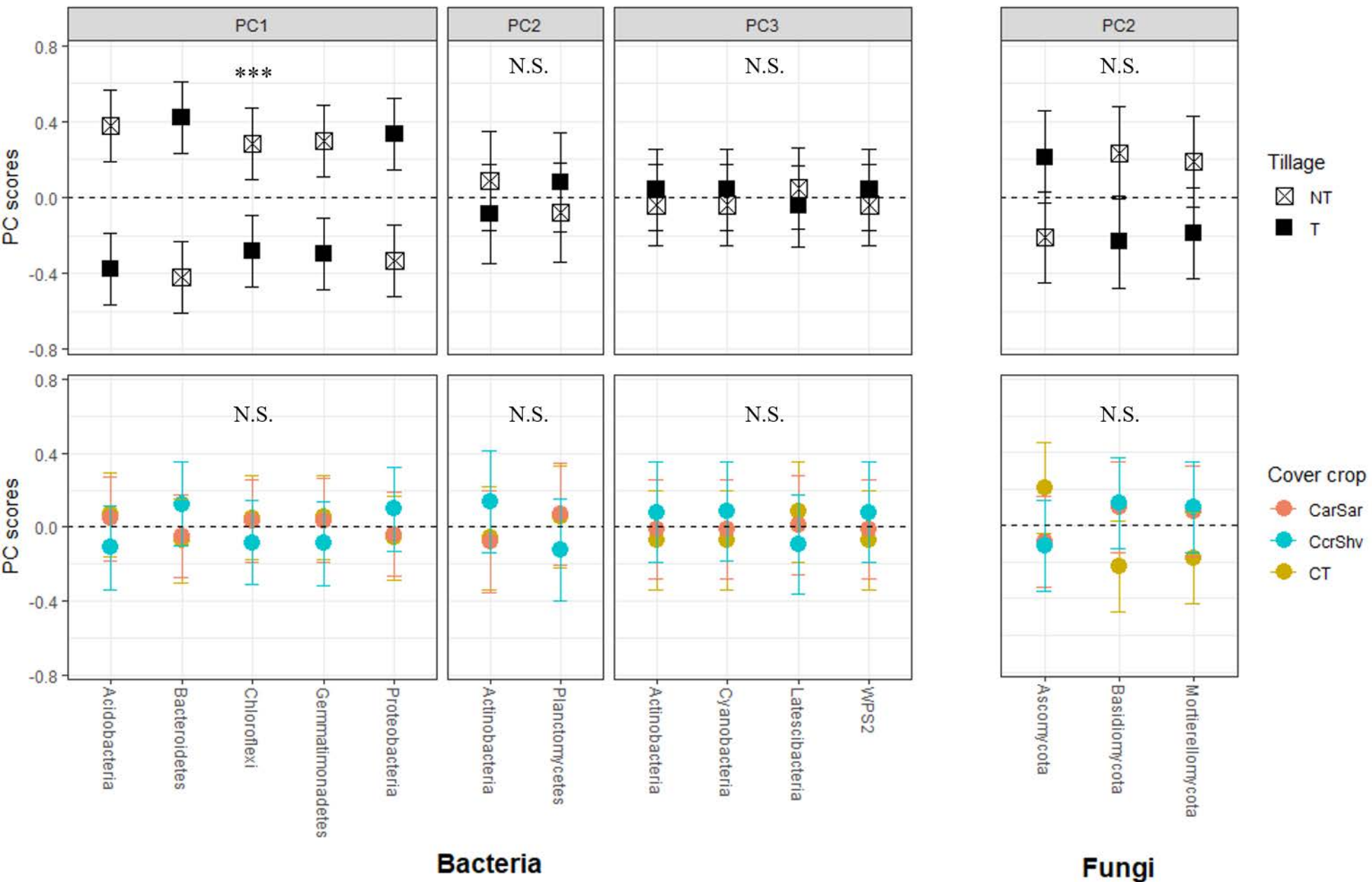

Supplement: Supplementary file 1 [file microorganisms-08-01773-s001.zip › Supp Figures.pdf]
